# Health economics study of paliperidone palmitate in the treatment of schizophrenia: a 12-month cohort study

**DOI:** 10.1186/s12888-024-05874-1

**Published:** 2024-06-12

**Authors:** Xing Luo, Fang Liu, Jin Lu, Yuqi Cheng, Xiufeng Xu, Xiaolin He, Yongbing Xia, Changqing Gao, Xian Xie, Yu Zhao, Chunqiang Gao, Hua Ding, Yuefei He, Lifen Zhang, Xi Zhang, Jianhui Song, Shunying Yang, Liming Liu, Wenming Chen, Wei Liu, Chuanlin Luo, Ensheng Pu, Ming Lei, Yan Wang, Zanzong Sun, Rucheng Yang, Yong Zhou, Xianrong Zhu, Bo Wang, Shuhua He, Donghua Gao, Zhongcai Li, Liqiong Huang, Tianlan Wang, Guangya Yang, Hong Liu, Jinkun Zhao, Jicai Wang

**Affiliations:** 1 Department of Psychiatry, First Affiliated Hospital of Kumming Medical University, Yunnan, China; 2 Yunnan Mental Health Center, Yunnan, China; 3 Yunnan Mental Hospital, Yunnan, China; 4 Zhaotong Mental Health Center, Yunnan, China; 5 The Third People’s Hospital of Qujing, Yunnan, China; 6 The Second People’s Hospital of Honghe Prefecture, Yunnan, China; 7 The Second People’s Hospital of Dali Prefecture, Yunnan, China; 8 The Second People’s Hospital of Yuxi, Yunnan, China; 9 The Second People’s Hospital of Pu’er City, Yunnan, China; 10 Baoshan Third People’s Hospital, Yunnan, China; 11 Dehongzhou Hospital of Traditional Chinese Medicine, Yunnan, China; 12 Xishuangbanna Mental Health Center, Yunnan, China; 13 Lijiang Second People’s Hospital, Yunnan, China; 14 The Second People’s Hospital of Chuxiong, Yunnan, China; 15 The Third People’s Hospital of Lincang, Yunnan, China; 16 The People’s Hospital of Wenshan, Yunnan, China

**Keywords:** Schizophrenia, Paliperidone palmitate, Economic benefits

## Abstract

**Background:**

To analyze the economic benefits of paliperidone palmitate in the treatment of schizophrenia.

**Methods:**

We collected 546 patients who met the diagnostic criteria for schizophrenia according to the 《International Statistical Classification of Diseases and Related Health Problems,10th》(ICD-10). We gathered general population data such as gender, age, marital status, and education level, then initiated treatment with paliperidone palmitate. Then Follow-up evaluations were conducted at 1, 3, 6, 9, and 12 months after the start of treatment to assess clinical efficacy, adverse reactions, and injection doses. We also collected information on the economic burden before and after 12 months of treatment, as well as the number of outpatient visits and hospitalizations in the past year to analyze economic benefits.

**Results:**

The baseline patients totaled 546, with 239 still receiving treatment with paliperidone palmitate 12 months later. After 12 months of treatment, the number of outpatient visits per year increased compared to before (4 (2,10) vs. 12 (4,12), Z=-5.949, *P* < 0.001), while the number of hospitalizations decreased (1 (1,3) vs. 1 (1,2), Z = 5.625, *P* < 0.001). The inpatient costs in the direct medical expenses of patients after 12 months of treatment decreased compared to before (5000(2000,12000) vs. 3000 (1000,8050), *P* < 0.05), while there was no significant change in outpatient expenses and direct non-medical expenses (transportation, accommodation, meal, and family accompanying expenses, etc.) (*P* > 0.05); the indirect costs of patients after 12 months of treatment (lost productivity costs for patients and families, economic costs due to destructive behavior, costs of seeking non-medical assistance) decreased compared to before (300(150,600) vs. 150(100,200), *P* < 0.05).

**Conclusion:**

Palmatine palmitate reduces the number of hospitalizations for patients, as well as their direct and indirect economic burdens, and has good economic benefits.

## Background

Antipsychotic medications remain the most fundamental and effective treatment for schizophrenia [1]. A major challenge in the treatment of schizophrenia is the poor adherence [2]. The relapse of the schizophrenia, increased hospitalization, frequent outpatient visits, prolonged treatment time, resulting in a significant increase in nursing costs, hindered the social integration of patients, continuous decline in social functioning, increased economic burden on patients’ families and society, and resulted in wastage of medical resources. Because of its extremely high relapse and disability rates, schizophrenia has become a major driver of the global disease burden [3], and it is particularly important to establish the effectiveness of early treatment and long-term patient compliance. The current domestic and international treatment guidelines recommend long-acting antipsychotic injections as an important strategy for preventing relapse, significantly improving the acceptance and compliance of patients with schizophrenia [4]. Once-monthly paliperidone palmite (PP1M), a second-generation antipsychotic long-acting injection, is the palmitate ester of risperidone (9-hydroxyrisperidone), the pharmacologically active metabolite of risperidone, recommended for the acute control of symptoms and maintenance treatment of schizophrenia [5]. The use of PP1M has simplified treatment regimens significantly due to its unique drug delivery method, thereby addressing patient adherence issues to a great extent. The continuous drug delivery provides a window for patient follow-ups, increases the frequency of patient and treatment team contact, and facilitates timely identification of missed doses, allowing for appropriate responsiveness. It also enables healthcare professionals to gain a certain understanding of changes in the patient’s condition [6]. The application of PP1M effectively inhibits the recurrence of symptoms, improves patients’ adherence and social functioning, and to a certain extent reduces the economic burden on patients and society [7]. Long-term mirror studies have shown that after initiating PP1M treatment, the annual cost of treatment for each patient has been decreasing, and the trend has been maintained [8]. Compared to long-acting formulations of olanzapine and risperidone, PP1M does not require any oral supplementation, with once-monthly dosing, the convenience of which means less administration time, costs, and fewer opportunities for non-adherence, thereby reducing the likelihood of patient relapse, lowering long-term inpatient care and emergency treatment medical expenses for patients [9]. Based on the above, PP1M has improved patient long-term adherence and disease stability from all aspects, reducing relapse rates and rehospitalization rates, and has a high cost-effectiveness. This study injected PP1M into real-world schizophrenia patients and followed up for 12 months to evaluate the economic benefits.

## Methods

### Study subject

The subjects of this study were drawn from 16 hospitals’ psychiatric outpatient or inpatient departments in various prefectures and cities in Yunnan Province. The period form October 2020 to October 2022.All patients and their families voluntarily accepted PP1M treatment and signed informed consent forms. This study was approved by the Ethics Committee of the First Affiliated Hospital of Kunming Medical University.

Inclusion criteria:: ① Meets the diagnostic criteria for schizophrenia according to ICD-10, with a PANSS (Positive and Negative Syndrome Scale) total score ≥ 70 points; ② Age range from 18 to 60, no restrictions on gender; ③ Patients covered by local effective basic medical insurance.

Exclusion criteria: ① Patients with refractory schizophrenia, i.e., patients who have not responded well to a full course of treatment with three antipsychotic drugs (at least two different chemical structures) in the past five years, or who are unable to tolerate the adverse effects of antipsychotic drugs, or whose condition still relapses or worsens even with adequate maintenance or preventive treatment [10];② Patients with serious organic diseases or medical history(severe damage to liver and kidney function, etc.); ③ Clinically significant electrocardiogram (ECG) abnormalities; ④ Patients using three or more psychiatric drugs in combination, or patients using clozapine in high doses (more than 400 mg); ⑤Patients with long-term drug abuse or alcohol dependence; ⑥ Patients with suicide attempts in the 12 months prior to enrollment, or those who had serious suicidal ideation when enrolled; ⑦ Patients known or suspected to be allergic or intolerant to any excipients of risperidone, paliperidone, or any formulation of risperidone and paliperidone, or patients with inadequate response to adequate trials of risperidone or paliperidone; ⑧ Patients with a history of malignant syndrome or delayed movement disorders due to antipsychotic medication; ⑨ Pregnant and breastfeeding women, or those who are unable to use effective contraception during the trial period, or have had a childbearing plan for the last one year; ⑩ Body Mass Index(BMI; ≥ 40 kg/m^2^ or ≤ 17 kg/m^2^.

Criteria for termination or withdrawal: ① QTc interval ≥ 470 ms for men or ≥ 480 ms for women, or QTc increase ≥ 60 ms in the follow-up ECG examination after enrollment; ② Serious adverse events or request for stopping treatment due to adverse events; ③ Withdrawn informed consent; ④ Non-pharmacological reasons, such as serious physical diseases; ⑤ Loss of visits; ⑥ Clinicians believe that it is inappropriate to continue the injection of long-lasting injections; ⑦ Others.

### Study design

General demographic information of the patients including age, gender, marital status, education, past history, family history was collected by a self-administered questionnaire. A questionnaire was used to collect information from the patient’s family about the patient’s economic burden in the last 12 months, including the number of outpatient visits, outpatient costs, number of hospitalizations, hospitalization costs, transportation costs, lodging costs, food and beverage costs spent at the clinic, economic losses due to the reduction of work time and work capacity caused by the disease, and the economic costs due to destructive behaviors, and the method of calculating the direct economic burden was direct medical expenses = outpatient fees + hospitalization fees, direct non-medical expenses = transportation fees paid for medical consultation + accommodation fees + food and beverage fees; indirect economic burden = economic losses due to reduced working hours and working capacity caused by the disease + economic costs due to vandalism [11]. Patients who have never used paliperidone or risperidone oral formulation, or risperidone long-acting injection, determine patient tolerance to paliperidone by administering paliperidone extended-release tablets or risperidone tablets orally for 3 days prior to initiating treatment with this product. Patients with prior use of paliperidone or risperidone oral formulation, or risperidone long-acting injection can inject directly. The initial treatment regimen is to inject PP1M 150 mg into the deltoid muscle on the first day, 100 mg into the deltoid muscle on the 8th day, then after month injected third, the dose within the range of 75–150 mg is selected based on the patient’s tolerance and effectiveness, the injection site can be the deltoid or gluteal muscle, and the treatment lasts for 12 months. The patients were followed up with face-to-face visits at months 1, 3, 6, 9, and 12, and the efficacy of the treatment was assessed by the PANSS, and the Treatment Emergent Symptom Scale (TESS) was used to assess the adverse efficacy. And collecting information of relapse rate and economic burden of the patients over the 12 months of treatment.

### Study instruments

(1) Self-administered questionnaire: including patient’s age, gender, marital status, education, past history, family history, patient’s economic burden in the last 12 months.

(2) Positive and Negative Syndrome Scale (PANSS) [12]: It consists of three subscales, including positive symptoms (such as excitement and hostility, hallucinations and delusions, conceptual disorganization and other 7 items), negative symptoms (such as emotional blunting, passive withdrawal, stereotyped thinking and other 7 items), and general psychopathological symptoms (such as anxiety and depression, attention disorders, preoccupation and other 16 items). Each item is scored from 1 to 7, where 1 means no symptom, and 7 means extremely severe symptom. The higher the score, the more severe the patient’s symptoms.

(3) Treatment Emergent Symptom Scale (TESS): This scale comprises 36 adverse reactions related to behavioral toxicity, the nervous system, the autonomic nervous system, and the cardiovascular system. Each symptom is evaluated based on its severity, its relationship to the medication, and the corresponding treatment measures.

### Statistical analysis

The data were analyzed using SPSS 26.0, with a significance level of α = 0.05. The Shapiro-Wilk test was used to check for normal distribution in the measured data at each visit point. Data that conformed to normal distribution were represented using mean ± standard deviation (𝑥̄±𝑠), and paired t-tests were used for comparison before and after treatment. For non-normally distributed measurement data, the median (M) with lower quartile (Q1) and upper quartile (Q3), [M (Q1, Q3)] was used, and Wilcoxon tests were used for comparison before and after treatment. For categorical data such as gender, marital status, and education, frequency and proportion were used for representation. The missing data in the sample are removed by deleting the individuals containing missing data. Although this approach may lead to the loss of important hidden information, but the proportion of missing values in this sample is relatively small, and deleting them will not have a significant impact on the results.

## Results

### Demographic information

The demographic characteristics of participants were detailed in Table 1.

**Table 1:** General demographics of patients at baseline

Characteristic	*N* = 546
Age (years), M(SD)	35.16(0.45)
Gender n(%)	
Male	376(68.90)
Female	170(31.10)
Marital status n(%)	
Unmarried	333(61.00)
Married	160(29.30)
Divorced/separated	53(9.70)
Educational level n(%)	
Below junior high school	377(69.00)
High school	131(24.00)
University and above	38(7.00)
Smoking n(%)	
Yes	359(65.80)
No	187(34.20)

### Changes in the number of outpatient visits and hospitalizations before and after treatment

After 12 months of treatment patients had more annual outpatient visits (Z=-5.949, *P*<0.001) and fewer hospitalizations (Z = 5.625, *P*<0.001) compared to pre-treatment in Table 2.

**Table 2:** Changes of the average number of outpatient visits and hospitalizations per year before and after treatment [M (QL, QU) times]

	base line	12th	Z	*P*
number of outpatient visits	4(2,10)	12(4,12)*	-5.949	<0.001
number of hospitalizations	1(1,3)	1(1,2)*	5.625	<0.001

Comparison with pre-treatment, *P < 0.05

### Comparison of patients’ economic burden (direct economic burden, indirect economic burden) before and after treatment

After 12 months of treatment, the direct medical costs of patient treatment in the hospitalization fee decreased compared with the pre-treatment, and the difference was statistically significant (Z = 3.531, *P* < 0.05), and there was no significant change in the outpatient fee (Z = 1.288, *P*>0.05) and the direct non-medical costs (transportation, accommodation, meals, family members’ accompanying expenses) (Z = 1.107, *P* > 0.05).

Patients’ indirect costs (lost wages for patients and families, financial costs due to vandalism, and costs of seeking non-medical help) decreased after 12 months of treatment compared to pre-treatment, and the difference was statistically significant (Z = 3.957, *p* < 0.05) in Table 3.

**Table 3:** Comparison of patients’ financial burden before and after treatment [M(QL, QU)]

	direct economic burden			indirect economic burden
	0utpatient costs	hospital costs	direct non-medical costs	
Base line	250(150,400)	5000(2000,12000)	202(100,355)	300(150,600)
12th	200(100,426)	3000(1000,8050)^*^	150(65, 371)	150(100,200)^*^
*Z*	1.288	3.531	1.107	3.957
*P*	0.198	<0.001	0.268	<0.001

Comparison with pre-treatment, *P < 0.05

## Discussion

The emergence of second-generation antipsychotics has significantly improved the efficacy of schizophrenia medications and reduced adverse effects [13]. However, there are still many difficulties in the treatment of schizophrenia, such as treatment resistance and poor response to medication in some patients, as well as low patient compliance and treatment interruption, leading to relapse and social function impairmen. In recent years, long-acting injections have been recommended for patients with poor compliance to improve long-term prognosis [14]. PP1M has been shown to be effective in controlling psychotic symptoms at an early stage, and because it only requires monthly injections, which greatly improves patient adherence, and its effectiveness in preventing relapse is superior to that of oral antipsychotics in terms of efficacy [15]. Numerous studies have demonstrated that PP1M improves patients’ adherence to treatment and reduces relapse rates in different ways [16, 17], And the decline in recurrence rates was strongly associated with a subsequent decline in hospitalisation rates and shorter hospital stays.

Consistent with previous studies [18], in this study, from the initiation of PP1M treatment to its conclusion, patients had an increase in the average number of outpatient visits per year and a decrease in the number of inpatient hospitalizations. PP1M treatment requires patients to attend an outpatient clinic once a month for injections, whereas patients treated with oral antipsychotics usually have routine visits every 3–6 months, so the number of visits increased in this study. It has been shown that patients on PP1M had significantly fewer outpatient visits per year on average for relapse of psychiatric symptoms compared to pre-treatment, and the increase in outpatient visits was all-cause [19]. Use of PP1M may reduce readmission rates in the long term [7]. The study also obtained consistent findings that the average annual number of patient hospitalisations was reduced with PP1M. In actual clinical practice, Taylor [20] conducted a 12-month prospective, non-interventional observational study showed that PP1M significantly reduced the number of hospitalizations and length of stay per patient per year. Other studies have yielded the same results, with the number of hospitalizations, hospital admissions, length of stay, and time in bed during hospitalization significantly reduced after 1 year of treatment with PP1M compared with pretreatment [21]. And fewer hospitalisations mean fewer inpatient and nursing costs.

The economic burden of schizophrenia is caused by direct healthcare costs, indirect productivity losses, and the financial cost of the accident and incident. In this study, patients’ economic burden (direct and indirect) decreased after 1 year of treatment compared with the pre-treatment period, with the decrease in direct economic burden driven by hospitalization and the decrease in indirect economic burden due to the reduction in home care costs after symptomatic improvement and the reduction in vandalism, which resulted in a reduction in the economic costs. On the other hand, patients come to outpatient clinics on time, the recurrence rate decreases, the average number of hospitalisations per year decreases, and healthcare resources are effectively allocated, thus improving the efficiency of healthcare resource allocation and reducing the consumption of healthcare resources [22], Further validation of the good economics of the PP1M. Long-term studies have shown that after initiating PP1M treatment, patients long-term inpatient care, emergency treatment, and home care, and the annual cost of treatment per patient is decreasing [8]. Previous studies have compared the cost of PP1M with other antipsychotics from different perspectives. From a healthcare system perspective, the total cost of PP1M was lower than that of risperidone long-acting injections [23]. Therefore, replacing risperidone long-acting injections with PP1M can reduce the burden and is a healthcare cost-saving strategy. From a payer perspective, PP1M has a higher cost-effectiveness with lower expected costs, recurrence rates, hospitalisation rates, emergency room visit rates, and higher quality-adjusted life years (QALYs) [23]. Therefore, PP1M has a high economic benefit when combining the patient’s readmission rate, crash rate, social functioning, and continued treatment at a later stage.

### Limitations

With a large sample size and a long observation period, the results of this study are informative, but there are some limitations. Firstly, the study did not include a control group, and thus can only be compared with existing historical results, rather than contemporary studies with random allocation. Secondly, during the PP1M treatment, some patients still received oral medications such as clozapine, olanzapine, lurasidone, and aripiprazole. The study did not analyze the drug dosages, which may have an impact on the research results.

## Conclusion

In conclusion, this study supports that PP1M improves treatment adherence in patients with schizophrenia, reduces the average number of hospitalisations per year, and reduces the financial burden of patients in different ways, which is economically beneficial and provides more options for the long-term treatment of schizophrenia.

## Data Availability

The datasets used and/or analyzed during the current study are available from the corresponding author on reasonable request.

## Abbreviations

ICD-10: The International Statistica Classification of Diseases 10th Revision
BMI: Body Mass Index
PANSS: Positive and Negative Syndrome Scale
PP1M: Once-monthly paliperidone palmite
TESS: Treatment Emergent Symptom Scale
